# Supplementary material for: Evaluation of connectivity map shows limited reproducibility in drug repositioning
Source: Sci Rep. 2021 Sep 2;11:17624. doi: 10.1038/s41598-021-97005-z (PMC8413422; doi:10.1038/s41598-021-97005-z)
Supplement: Supplementary file 4 — Supplementary Information 4. [file 41598_2021_97005_MOESM4_ESM.pdf]

Evaluation of Connectivity Map shows limited reproducibility in drug repositioning

Nathaniel Lim[1,3], *Paul Pavlidis[2,3]

1. Genome Science and Technology Graduate Program, University of British Columbia, Vancouver, BC V6T1Z4, Canada.
2. Department of Psychiatry, University of British Columbia, Vancouver, BC V6T1Z4, Canada.
3. Michael Smith Laboratories, University of British Columbia, Vancouver, BC V6T1Z4, Canada.

[File: SuppTable_S1.CSV]

Supplementary Table S1: Combinations of compounds, concentrations and cell lines that are common between both CMap 1 and 2 (“Harmonized data”). The treatment duration for all combinations is fixed at 6 hours.

[File: SuppTable_S2.CSV]

Supplementary Table S2: Combinations of compounds and cell lines used as signatures from CMap 1 to perform L1000-Query on CMap 2 data; strongest compound concentrations are used. The treatment duration for all combinations is fixed at 6 hours and all compounds are part of CMap 2’s Touchstone data subset.

[File: SuppTable_S3.CSV]

Supplementary Table S3: Combinations of compounds and cell lines used as signatures from CMap 1 to perform L1000-Query on CMap 2 data. The treatment duration for all combinations is fixed at 6 hours and all compounds are part of CMap 2’s Touchstone data subset. Unlike Supplementary Table S2, the signatures used in Supplementary Table S3 are from the harmonized data. For the “Hybrid Threshold” case (see Methods), the signatures “10µM rosiglitazone in MCF7” and “10µM scopolamine in PC3” were excluded due to inadequate number of differentially expressed genes.

Chemical Compound	CMap 1 Concentration (µM)	CMap 2 Concentration (µM)	De Abrew et al. Concentration (µM)
GENISTEIN	10.0	10.0	10.0
METFORMIN	10.0	10.0	10.0
PHENFORMIN	10.0	10.0	10.0
TROGLITAZONE	10.0	10.0	10.0
VORINOSTAT	10.0	10.0	10.0
CHENODEOXYCHOLIC-ACID	10.2	10.0	10.0
GRISEOFULVIN	11.2	10.0	10.0
METHOTREXATE	8.8	10.0	10.0
CLOBETASOL	8.6	10.0	10.0
KETOCONAZOLE	7.6	10.0	10.0
PROGESTERONE	12.8	10.0	10.0
FLUTAMIDE	14.4	10.0	10.0

Supplementary Table S4: Compounds and concentrations used in the comparison of CMap 1 and 2 against the De Abrew dataset. The treatment duration for all compounds is fixed at 6 hours and the cell line used is MCF7. With the exception of the first five compounds (genistein, metformin, phenformin, troglitazone and vorinostat), we chose conditions that have a compound concentration of between 5 - 15µM in CMap 1 (as the corpus lacks similar conditions with exactly 10µM).

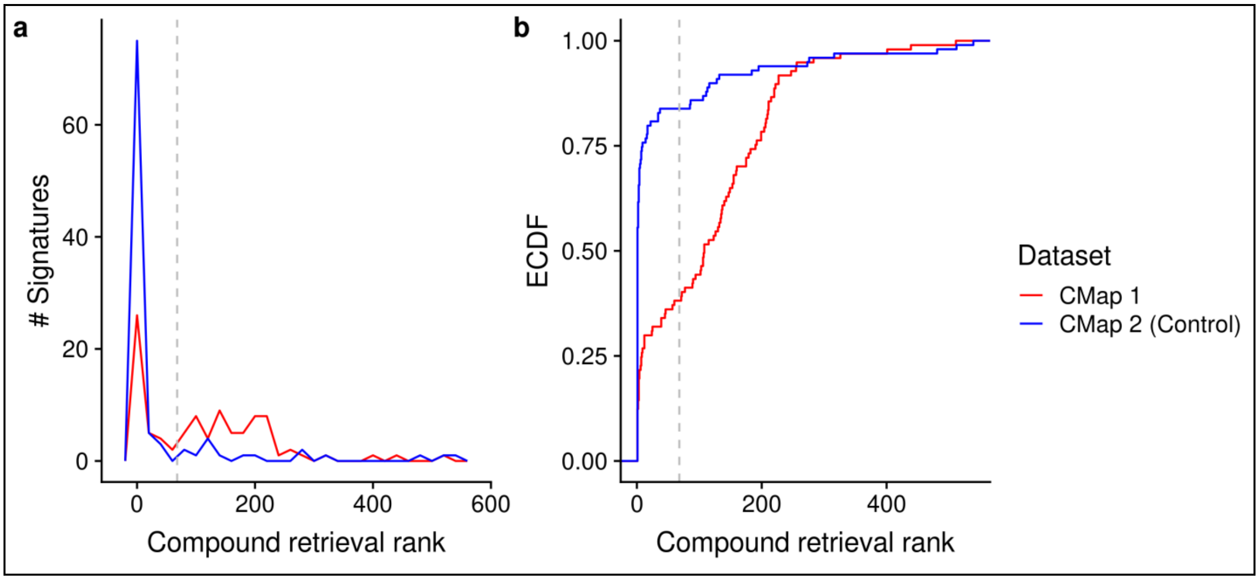

Supplementary Figure S1: Distribution of compound retrieval ranks (1 is best) from querying CMap 2 using signatures derived from CMap 1 (red lines; “Hybrid Threshold”) or CMap 2 (blue lines; self-query) data, as in main Figure 1, but with the added constraint that the conditions of the signatures are from the “harmonized data” subset (Supplementary Table S3, N = 97). Panel “a” shows the distribution while “b” is the empirical cumulative distribution function (ECDF) of the same results. The dotted grey line indicates the profiles where the query compound is ranked in the top 10% (rank $\leqslant$ 68).

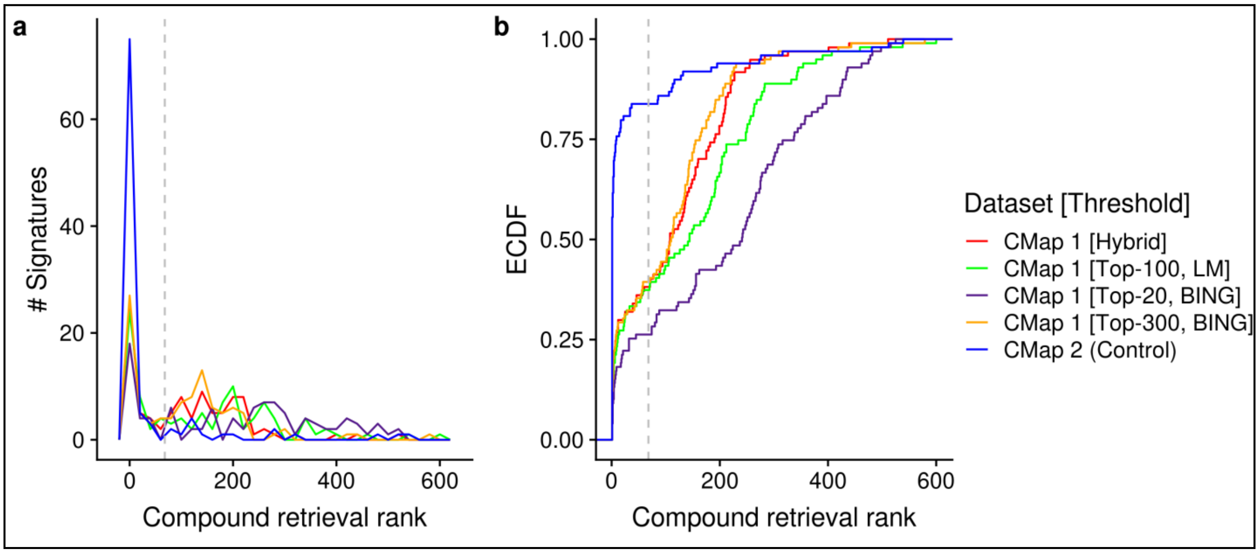

Supplementary Figure S2: Distribution of compound retrieval ranks (1 is best) from querying CMap 2 using signatures derived from CMap 1 (non-blue lines) or CMap 2 (blue lines) data, as in Supplementary Figure S1; the various CMap 1 signatures are derived using different thresholding methods (See Methods; Supplementary Table S3, N = 97). Panel “a” shows the distribution while “b” is the empirical cumulative distribution function (ECDF) of the same results. The dotted grey line indicates the profiles where the query compound is ranked in the top 10% (rank $\leqslant$ 68).

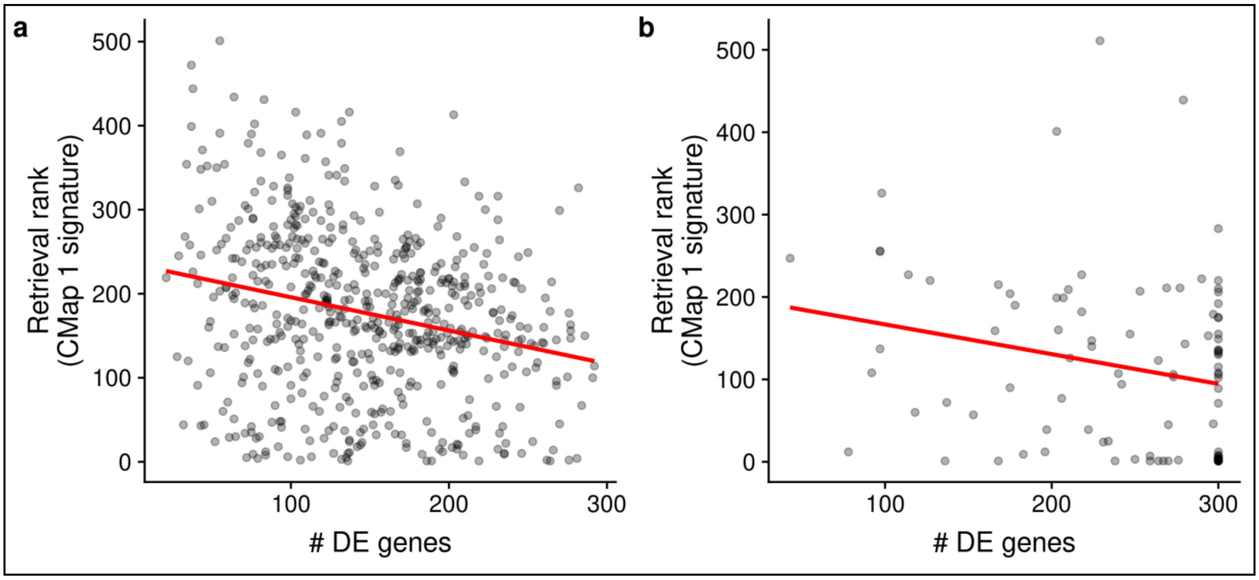

Supplementary Figure S3: Scatter-plot of compound retrieval ranks from querying CMap 2 using signatures derived from CMap 1 data against total number of DE genes (in the CMap 1 signatures). Linear regression of the data is shown in red. The underlying data in panel "a" is from the 588 query signatures of the strongest concentration (Supplementary Table S2); "b" is from the 97 query signatures from the harmonized data (i.e. common concentration) using the "Hybrid Threshold" (Supplementary Table S3). The Spearman correlations between retrieval ranks and total number of DE genes are -0.24 and -0.26 for the data in "a" and "b" respectively.

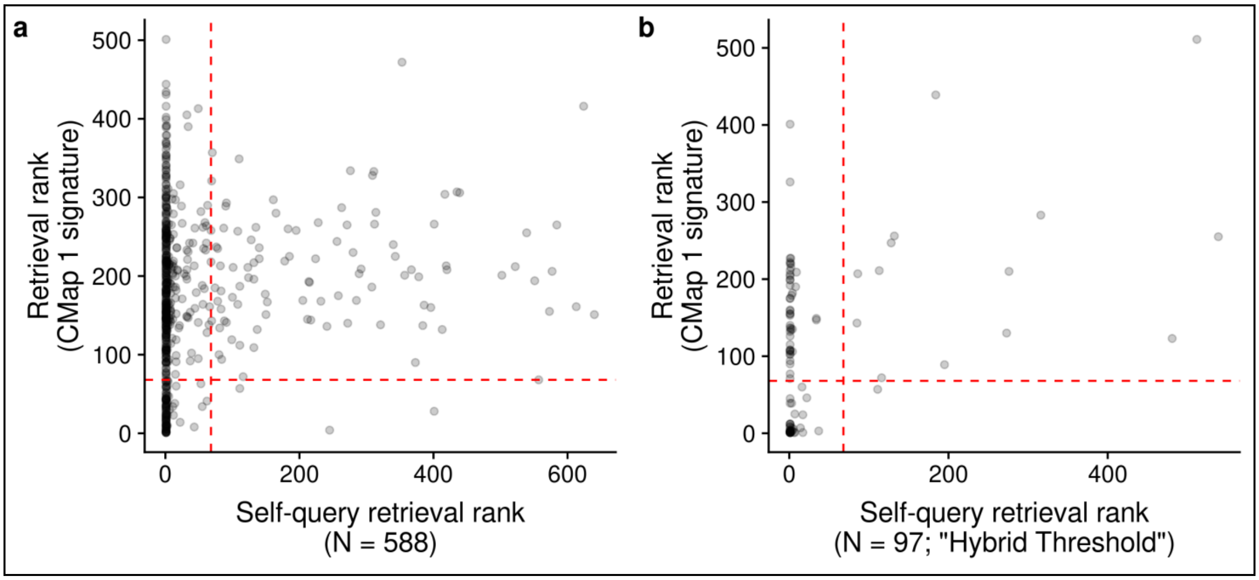

Supplementary Figure S4: Scatter-plot of compound retrieval ranks from querying CMap 2 using CMap 1 data-derived signatures against those of CMap 2 self-queries. The dotted red lines indicate the profiles where the query compound is ranked in the top 10% (rank ⩽ 68). The underlying data in panel "a" is from the 588 query signatures of the strongest concentration (Supplementary Table S2); "b" is from the 97 query signatures from the harmonized data (i.e. common concentration) using the "Hybrid Threshold" (Supplementary Table S3).

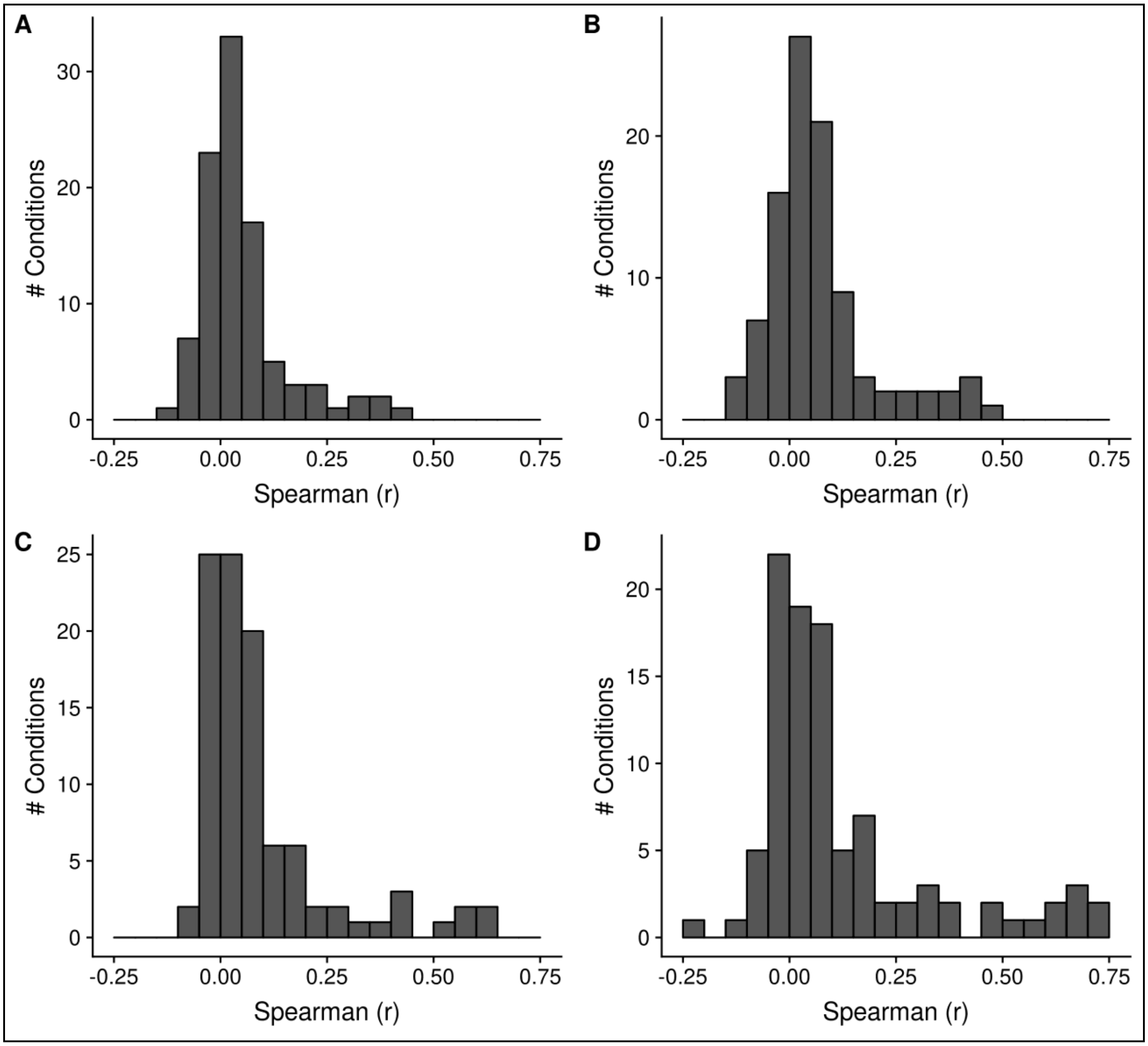

Supplementary Figure S5: Distribution of pairwise rank correlation of DE profiles from CMap 1 against CMap 2; profiles are limited to compounds in the “Touchstone” subset. Panels “a” and “c” are comparisons against CMap 2-FC; “b” and “d’ against CMap 2-MZS. All genes common between CMap 1 and CMap 2 are used in “a” and “b”, while only “landmark genes” are used in “c” and “d”. (N = 98 conditions).

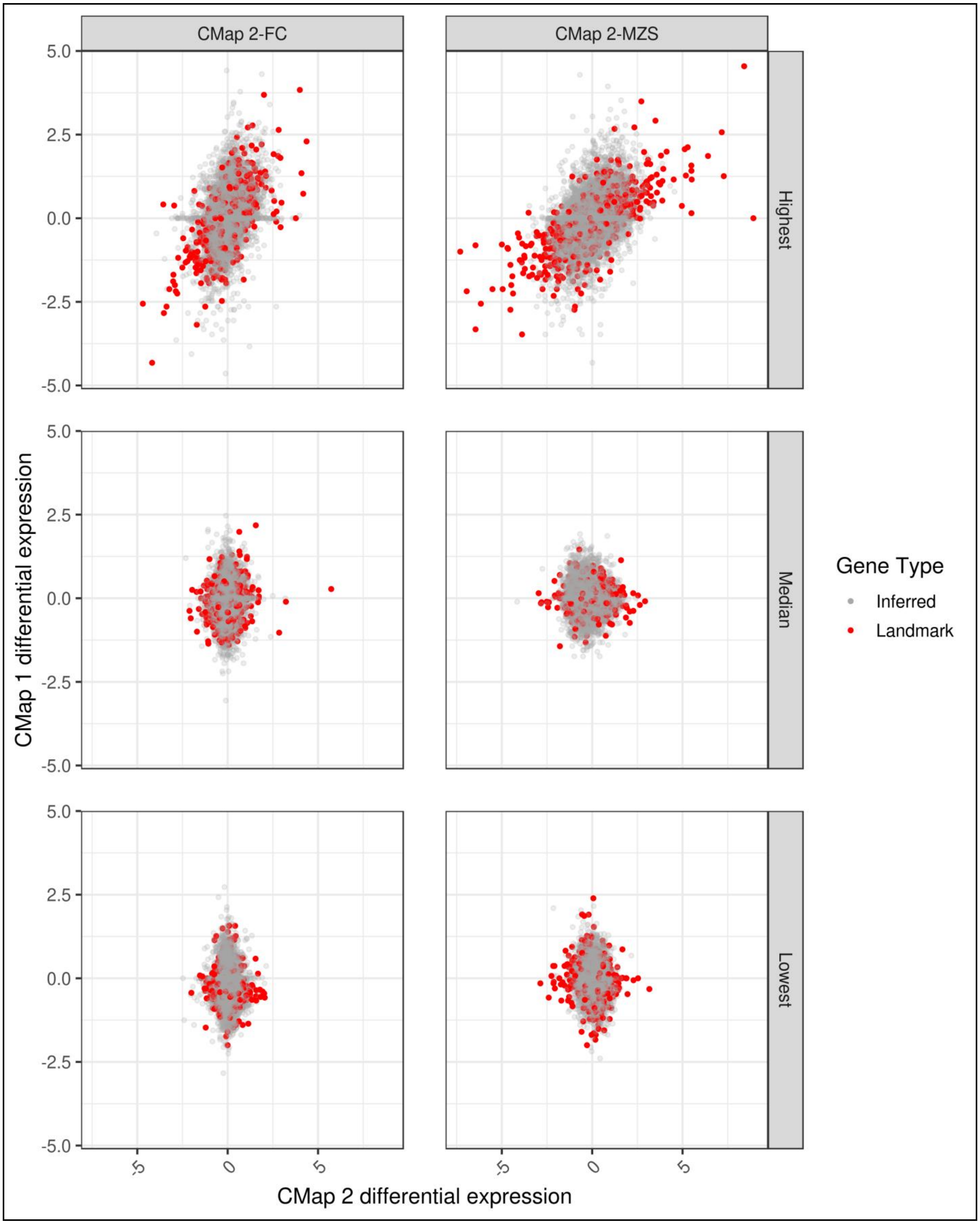

Supplementary Figure S6: Representative scatter-plots of gene DE values for six different pairs of profiles with different levels of between-CMap agreement (CMap 1 – CMap 2; high, medium or low). Each point represents one gene. The x-axis are values derived from CMap 2 profiles and y-axis are from CMap 1. The plots from top-to-bottom are of profiles with the highest, median and lowest pairwise rank correlation between CMaps respectively; plots on the left use the fold change version of CMap 2 (CMap

2-FC) while those on the right use the moderated Z-score version (CMap 2-MZS). Genes where the expression level is measured in CMap 2 (“Landmark genes”) are coloured red while those that are inferred are in grey. In order from top-to-bottom and from left-to-right, the profiles are (values in bracket are rank correlation of landmark genes): 1µM trichostatin-A in MCF7 (r = 0.63), 10µM vorinostat in MCF7 (r = 0.72), 10µM 16,16-dimethylprostaglandin-E2 in PC3 (r = 0.03), 10µM SR-95639A in PC3 (r = 0.04), 10µM orlistat in PC3 (r = -0.10) and 10µM resveratrol in MCF7 (r = -0.22). Treatment time for all profiles was 6 hours.

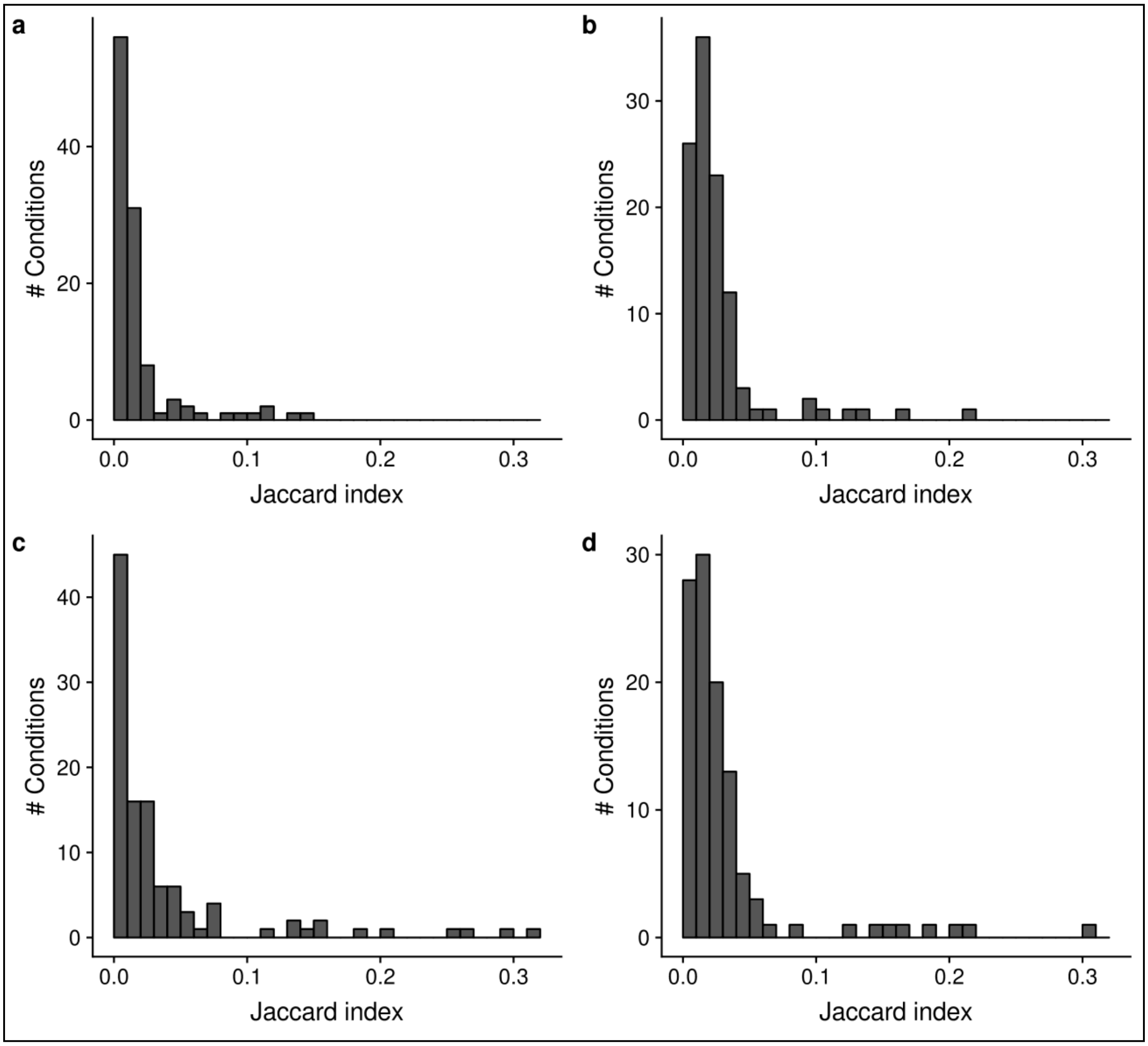

Supplementary Figure S7: Distribution of pairwise Jaccard index of DE profiles from CMap 1 against CMap 2; underlying data is similar to main Figure 2, but using a different similarity metric. Panels “a” and “c” are comparisons against CMap 2-FC; “b” and “d” against CMap 2-MZS. All genes common between CMap 1 and CMap 2 are used in “a” and “b’, while only “landmark genes” are used in “c” and “d”. (N = 109 conditions).

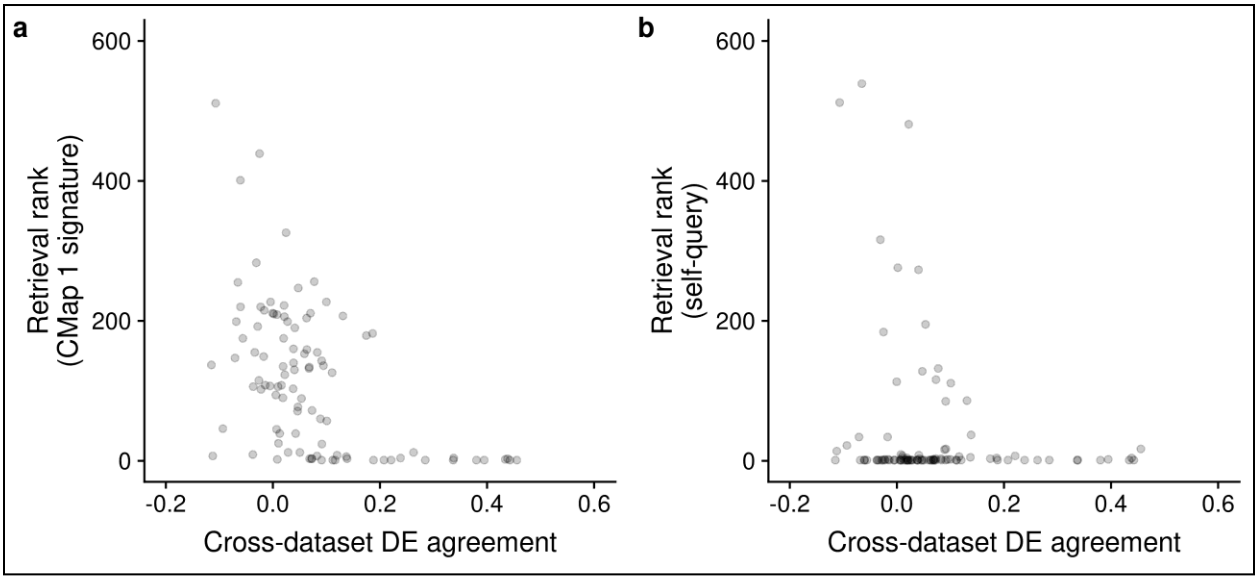

Supplementary Figure S8: Scatter-plot of compound retrieval ranks from querying CMap 2 with signatures derived from CMap 1 or CMap 2 data (y-axis; Panels "a" and "b" respectively) against cross-dataset DE agreement (between CMap 1 and CMap 2-MZS; N = 97); the cross-dataset agreement values reported here are calculated using rank correlation on all genes.

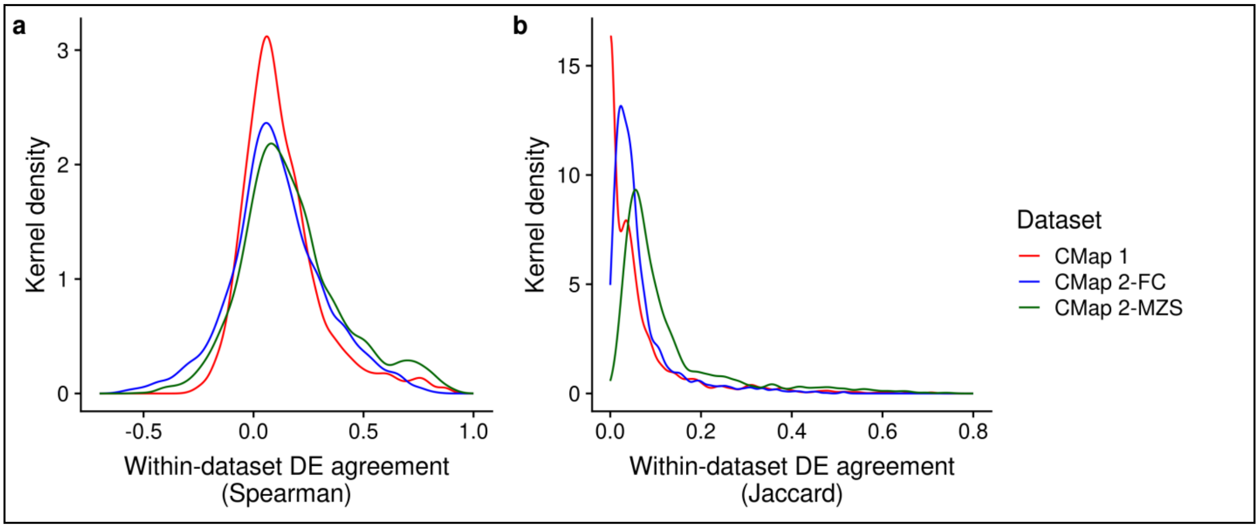

Supplementary Figure S9: Distribution of maximum ("best case") pairwise similarity values ("a": rank correlation, "b": Jaccard index) for DE profile replicates of the same condition calculated using landmark genes, within each dataset. The number of unique conditions assessed for CMap 1, CMap 2-FC and CMap 2-MZS are 1614, 2416 and 2477 respectively.

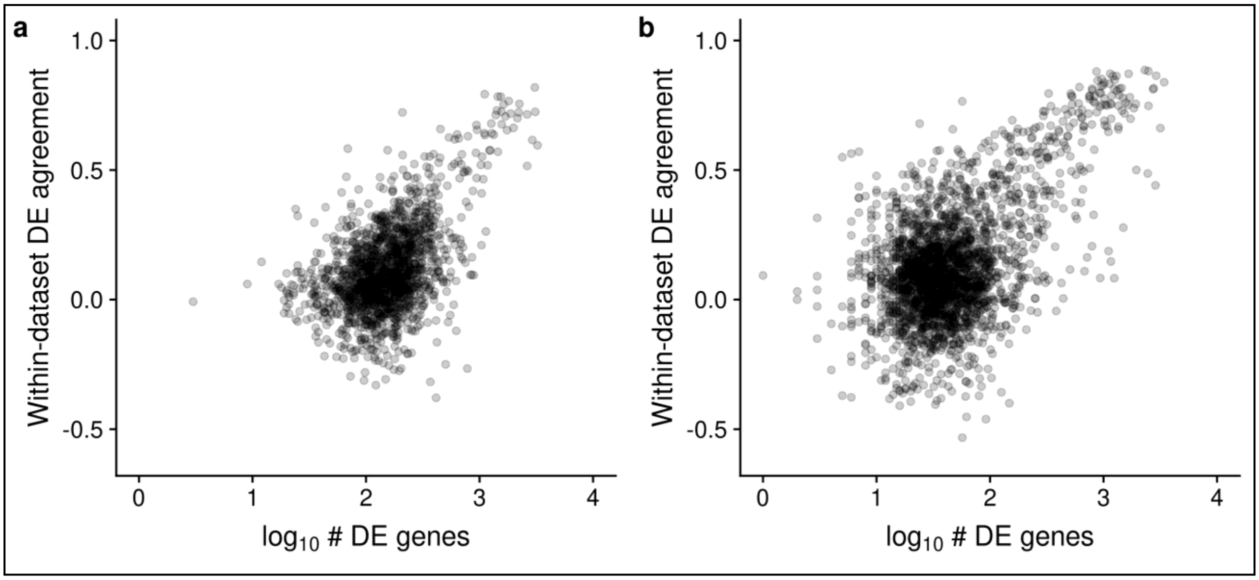

Supplementary Figure S10: Scatter-plot of within-dataset DE agreement ("a": CMap 1, "b": CMap 2-MZS) against the $\log_{10}$-transformed number of DE genes of the replicate pair; only the maximum ("best case") rank correlations for each unique condition/pair is used; for the number of DE genes, the lesser ("limiting case") of the two replicates in the comparison pair is used. The number of conditions assessed for CMap 1 and CMap 2-MZS are 1614 and 2477 respectively.

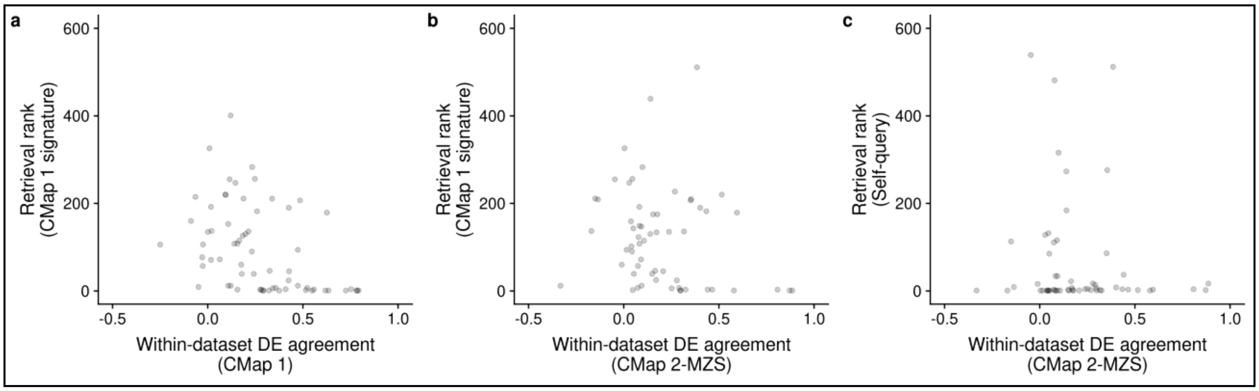

Supplementary Figure S11: Scatter-plot of compound retrieval ranks from querying CMap 2 with signatures derived from CMap 1 (y-axis; "a" and "b") or CMap 2 ("c") data against the within-dataset DE agreement (x-axis; CMap 1 for "a", CMap 2-MZS for "b" and "c"). As with Supplementary Figure S10, only the maximum rank correlation for each unique condition is shown for the within-dataset agreement. Total number of conditions shown in "a" is 65; for "b" and "c" are 58.

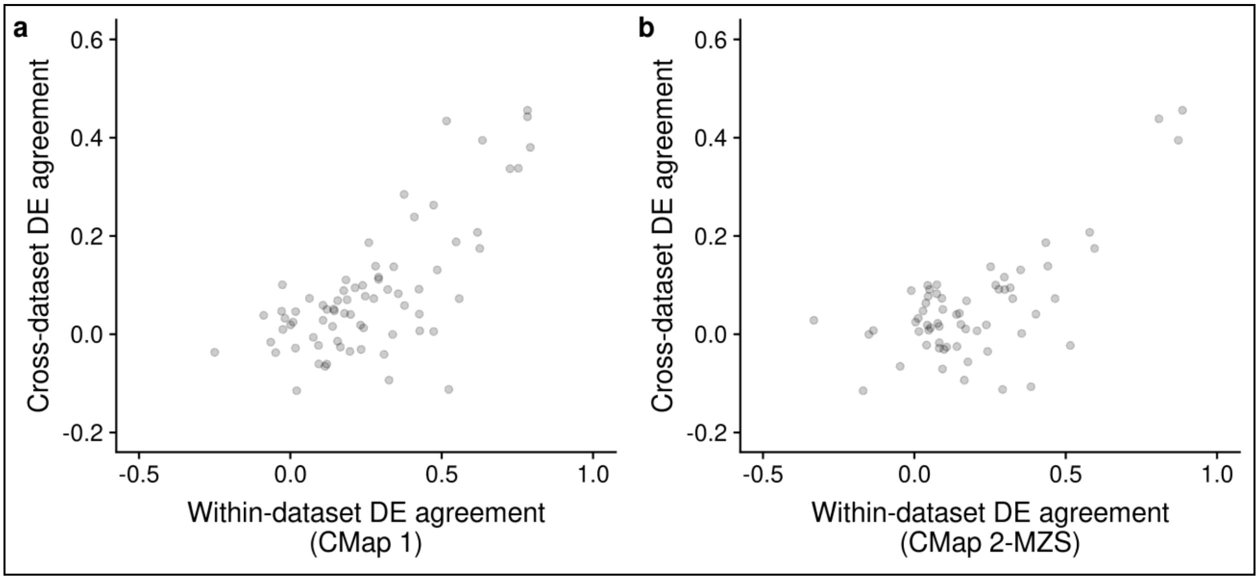

Supplementary Figure S12: Scatter-plot of cross-dataset DE agreement (between CMap 1 and CMap 2-MZS) against the within-dataset DE agreement ("a": CMap 1, "b": CMap 2-MZS). As with Supplementary Figure S10, only the maximum rank correlation for each unique condition is shown for the within-dataset agreement. Total number of conditions shown in "a" and "b" are 72 and 61 respectively.

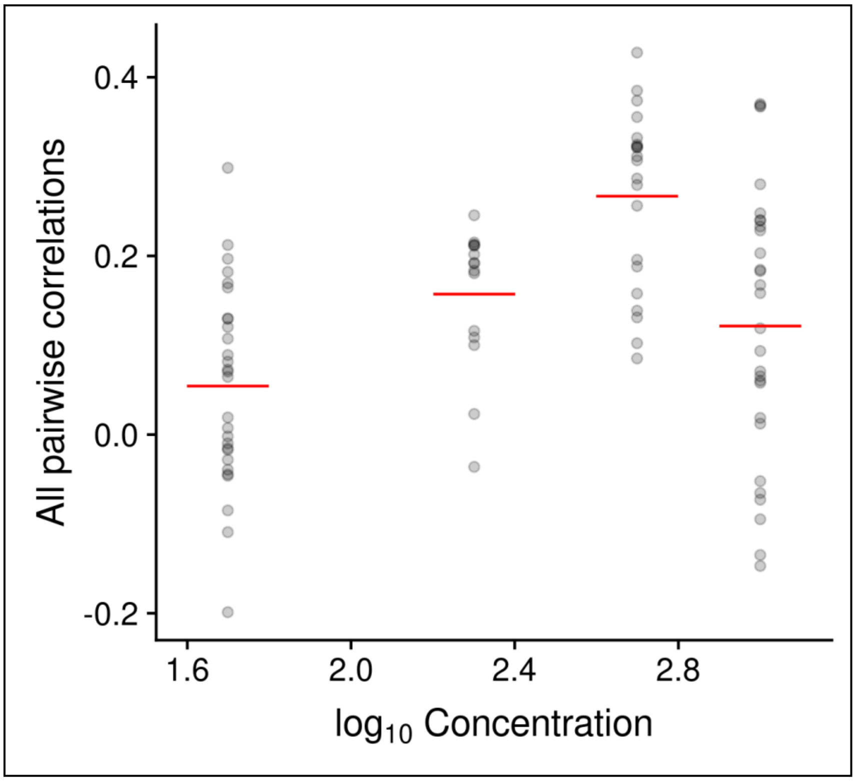

Supplementary Figure S13: Scatter-plot of all pairwise rank correlations of DE profiles against compound concentration, for valproic acid. Underlying data used is CMap 1, treated cell line is MCF7 and all genes were used in the calculations. Red lines indicate the mean correlation for each concentration group.

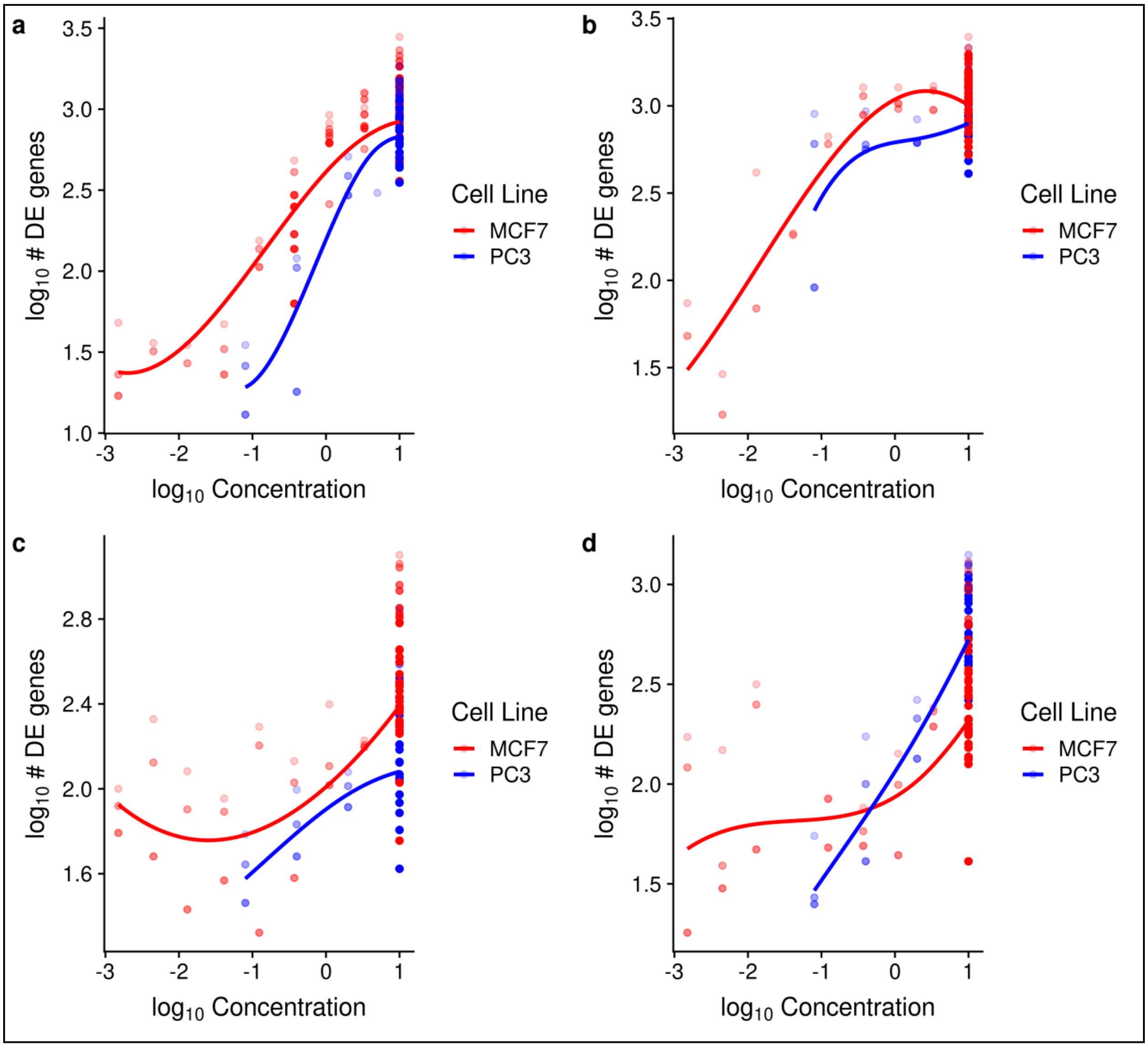

Supplementary Figure S14: Scatter-plot of DE "strength" of profile replicate pairs against compound concentration, for four different compounds (vorinostat, trichostatin A, geldanamycin and wortmannin for panels "a-d" respectively); DE "strength" refers to the $\log_{10}$-transformed number of DE genes of the replicate pair (as used in Supplementary Figure S10). Underlying data used is CMap 2-MZS and all genes were used in the calculations. Lines are LOESS fit of the values, while colours distinguish the cell lines being tested.

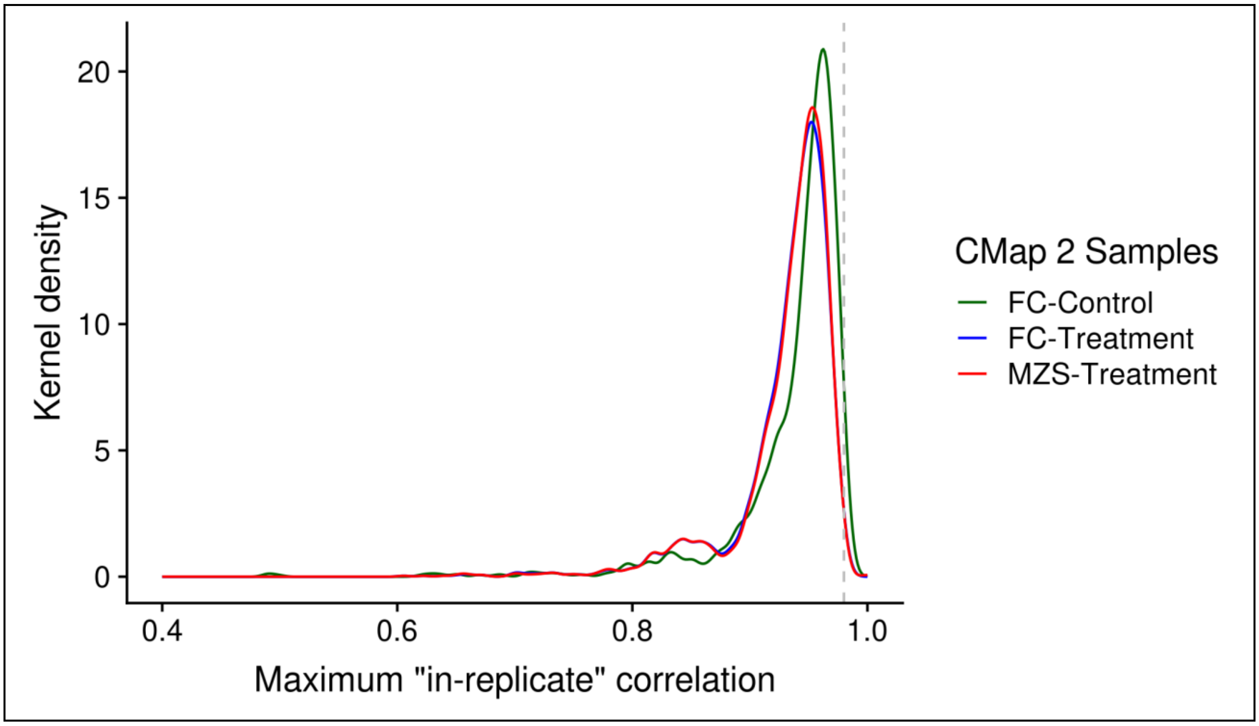

Supplementary Figure S15: Distribution of rank correlations of maximum "in-replicate" sample gene expression ("best case") for the DE replicate pairs used in main Figure 3, calculated using all genes. Unlike main Figure 5 in which the plotted "in-replicate" sample correlations are from the DE replicate pairs with the highest within-dataset DE agreement for each unique condition (i.e. main Figure 3), the sample correlations plotted here are not constrained by the performance of the DE pair. The number of unique conditions/pairs assessed for CMap 2-FC and CMap 2-MZS are 2416 and 2477 respectively. The dotted grey line indicates the typical median rank correlation of sample gene expression for intra-platform cross-laboratory comparisons reported in Chen et al. (2007, $r_s = 0.98$).

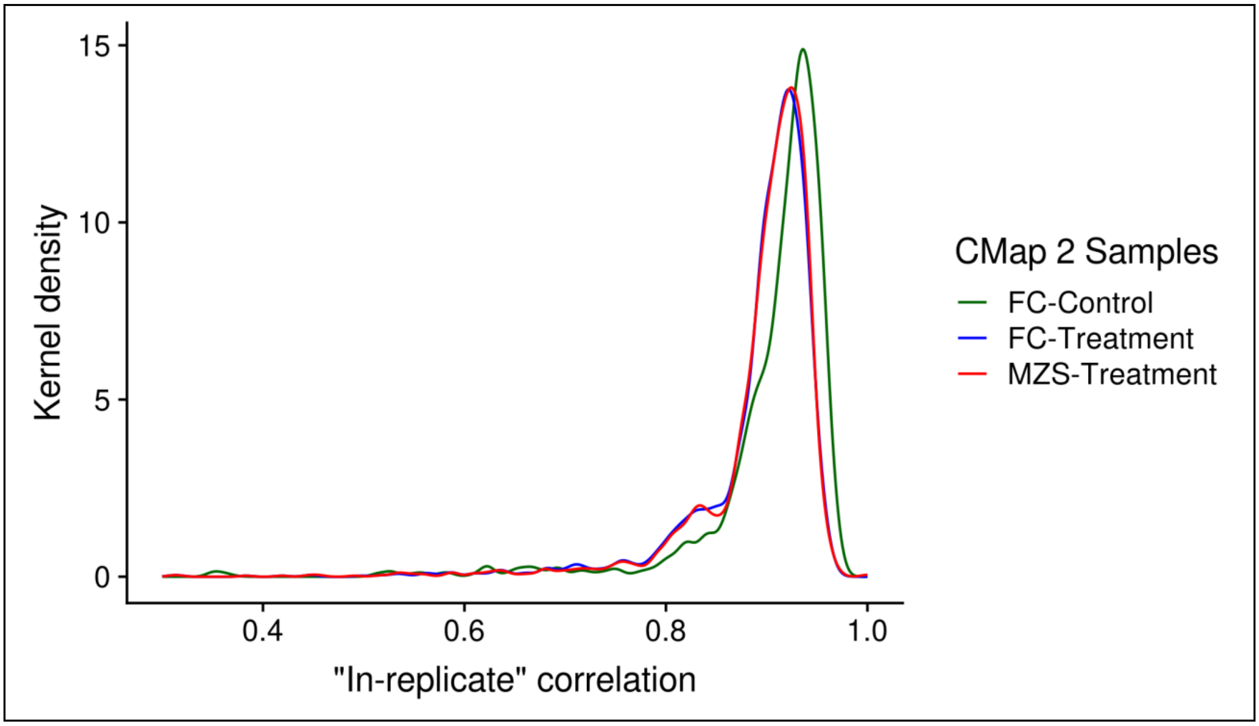

Supplementary Figure S16: Distribution of rank correlations of "in-replicate" sample gene expression for the DE replicate pairs used in main Figure 3, calculated using landmark genes only. The number of unique conditions/pairs assessed for CMap 2-FC and CMap 2-MZS are 2416 and 2477 respectively.

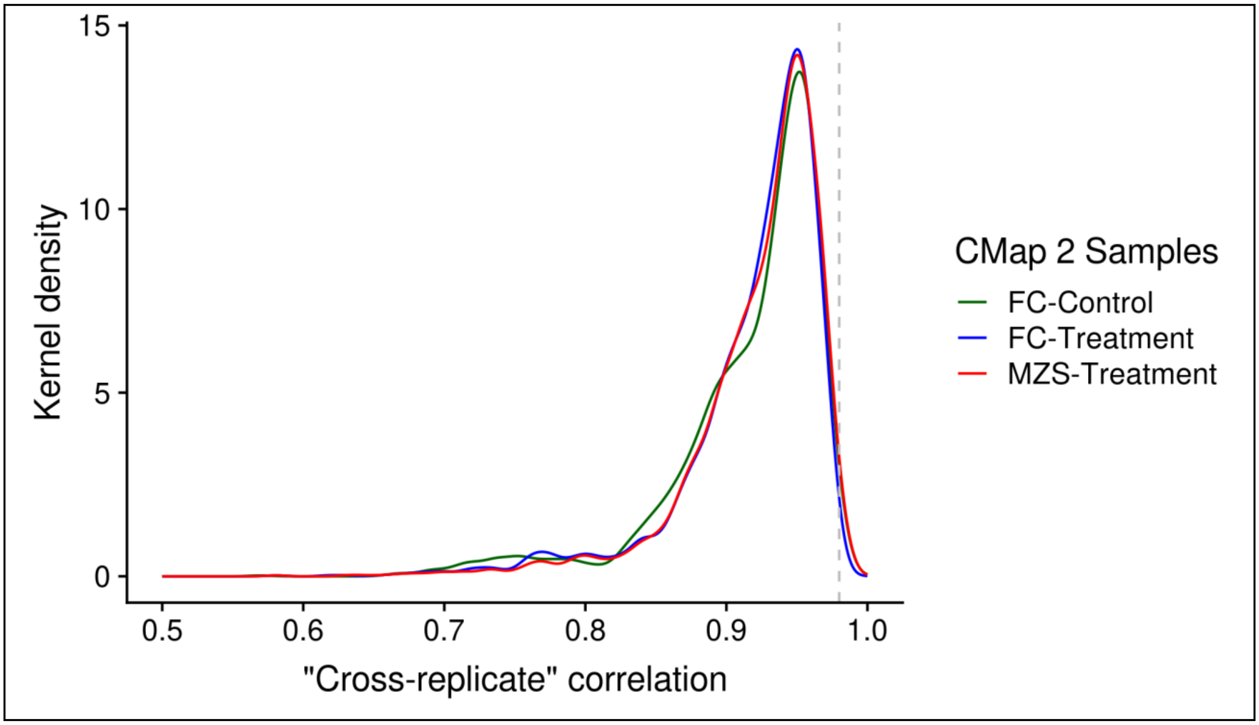

Supplementary Figure S17: Distribution of rank correlations of "cross-replicate" sample gene expression for the DE replicate pairs used in main Figure 3, calculated using all genes. The number of unique conditions/pairs assessed for CMap 2-FC and CMap 2-MZS are 2416 and 2477 respectively. The dotted grey line indicates the typical median rank correlation of sample gene expression for intra-platform cross-laboratory comparisons reported in Chen et al. (2007, $r_s = 0.98$).

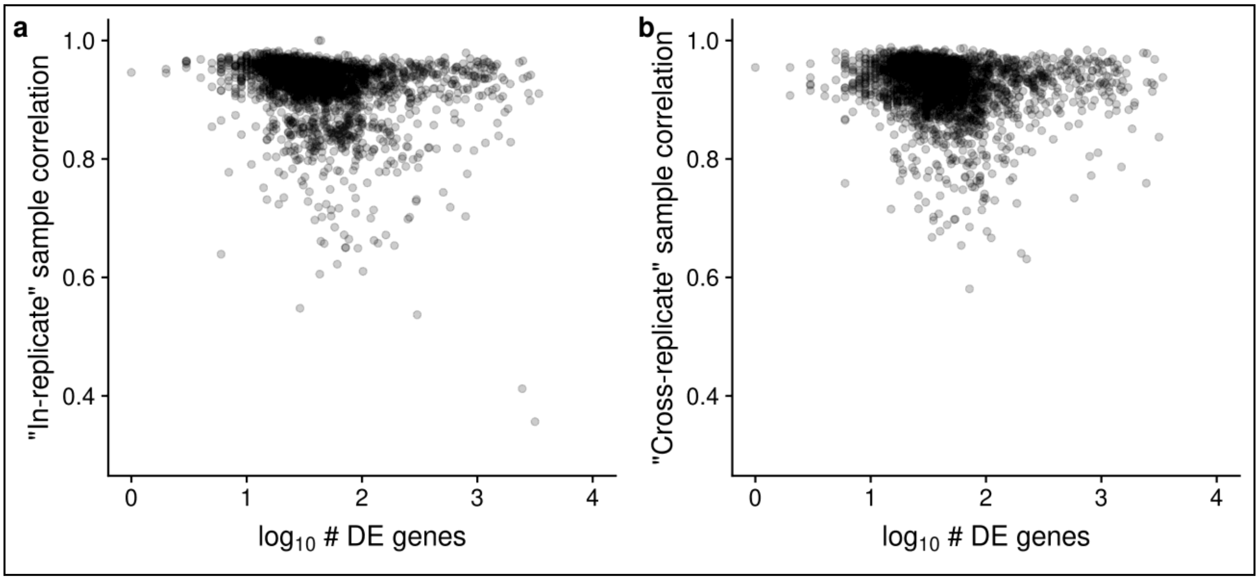

Supplementary Figure S18: Scatter-plot of within-dataset agreement of sample-level gene expression ("in-replicate" and "cross-replicate" for "a" and "b" respectively) against the $\log_{10}$-transformed number of DE genes of the replicate pair; as with

Supplementary Figure S10, the lesser number of DE genes between the replicates of a unique condition is used. Underlying data used is CMap 2-MZS and the number of unique conditions assessed is 2477.

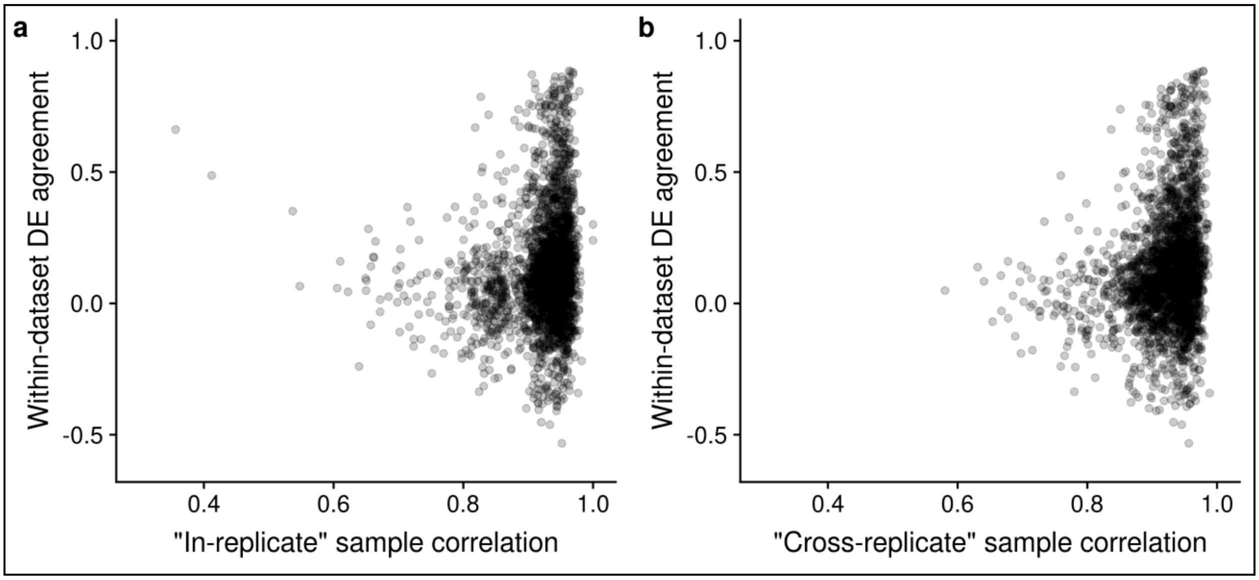

Supplementary Figure S19: Scatter-plot of within-dataset DE agreement against within-dataset agreement of sample-level gene expression ("in-replicate" and "cross-replicate" for "a" and "b" respectively). Underlying data used is CMap 2-MZS and the number of unique conditions assessed is 2477.

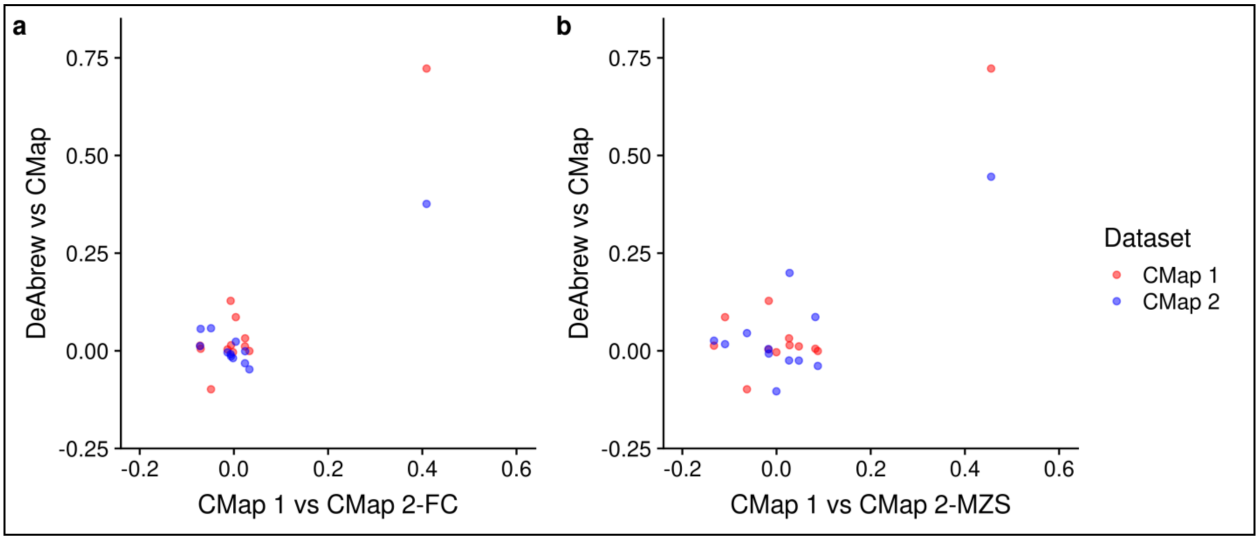

Supplementary Figure S20: Scatter-plot of DE reproducibility between the De Abrew dataset and CMap (y-axis; CMap 1 = red dots, CMap 2 = blue dots) against cross-dataset DE reproducibility between CMap 1 and CMap 2 (x-axis). The underlying versions of CMap 2 used in "a" and "b" are CMap 2-FC and CMap 2-MZS respectively. Each data point is 1 of the 12 compounds used to compare between De Abrew to CMap 1 to CMap 2 (Supplementary Table S4).
